# The neonicotinoid insecticide Clothianidin adversely affects immune signaling in a human cell line

**DOI:** 10.1038/s41598-017-13171-z

**Published:** 2017-10-18

**Authors:** Gennaro Di Prisco, Marco Iannaccone, Flora Ianniello, Rosalba Ferrara, Emilio Caprio, Francesco Pennacchio, Rosanna Capparelli

**Affiliations:** Department of Agricultural Sciences, University of Napoli “Federico II” – Via Università 100, 80055 Portici, Napoli, Italy

## Abstract

Clothianidin is a widely used neonicotinoid insecticide, which is a potent agonist of the nicotinic acetylcholine receptor in insects. This neurotoxic compound has a negative impact on insect immunity, as it down-regulates the activation of the transcription factor NF-κB. Given the evolutionary conserved role of NF-κB in the modulation of the immune response in the animal kingdom, here we want to assess any effect of Clothianidin on vertebrate defense barriers. In presence of this neonicotinoid insecticide, a pro-inflammatory challenge with LPS on the human monocytic cell line THP-1 results both in a reduced production of the cytokine TNF-α and in a down-regulation of a reporter gene under control of NF-κB promoter. This finding is corroborated by a significant impact of Clothianidin on the transcription levels of different immune genes, characterized by a core disruption of TRAF4 and TRAF6 that negatively influences NF-κB signaling. Moreover, exposure to Clothianidin concurrently induces a remarkable up-regulation of NGFR, which supports the occurrence of functional ties between the immune and nervous systems. These results suggest a potential risk of immunotoxicity that neonicotinoids may have on vertebrates, which needs to be carefully assessed at the organism level.

## Introduction

Neonicotinoids are among the most widely used insecticides in agriculture, which are effective at low dosage and show poor affinity for the nicotinic acetylcholine receptor of mammalian species^1,2^. The limited impact on non-target higher animals is, however, challenged by a growing number of studies, which support a negative effect of these systemic and persistent insecticides on several non-target organisms and ecosystem services^3,4^. In particular, pollinators seem to be particularly affected. Indeed, in spite of the fact that acute lethal effects are rarely observed^5^, there are a number of reports on sub-lethal effects, such as impaired honeybee learning or homing behavior^6–8^, and a stronger impact on pollinators of various pathogens^9–13^. This latter effect is in part due to the immunosuppressive action exerted by neonicotinoids^14,15^ which further exacerbates the negative impact that viral pathogens and *Varroa destructor* have on honeybee defense barriers^16–19^.

The molecular mechanism underlying the negative effect of the neonicotinoid Clothianidin on insect immune response has been recently reported^14^. Basically, this insecticide is able to exert a negative effect on the activation of the nuclear factor-κB (NF-κB) and of the downstream immune barriers, which promotes uncontrolled viral replication in honeybees bearing covert infections^14^. Moreover, other immune responses controlled by this transcription factor, both cellular and humoral, are down-regulated by neonicotinoids^15^, suggesting the occurrence of a wider impact of these insecticides on immunity.

NF-κB has a central role in the immune response by animals^20^, and, therefore, any defense pathway, conserved across distant evolutionary lineages, under control of this transcription factor could be influenced by a shared negative regulation of its activation. This could account for the proposed link between the use of neonicotinoids and the increasing incidence of pathologies in different animal groups^4,19^. It does not require a leap of imagination to speculate that neonicotinoids may have possible negative effects on human health, by similarly interfering with the regulation of the immune system. This is a hypothesis that certainly merits to be investigated, as part of a more comprehensive effort towards a thorough characterization of neonicotinoid impact on human health, which, surprisingly, is still in its infancy^21,22^.

Here we contribute to fill this gap of knowledge, by focusing our attention on the impact of the neonicotinoid Clothianidin on the human immune response, using an *in vitro* model system to characterize the effects that this molecule has on gene expression profile upon immune challenge. This has been done by RNA sequencing in the monocytic human cell line THP-1, as affected by Clothianidin exposure, and by studying how this latter can influence pro-inflammatory cytokine release upon immune challenge.

## Results

### Clothianidin disrupts NF-κB  signaling

**Figure 1:**
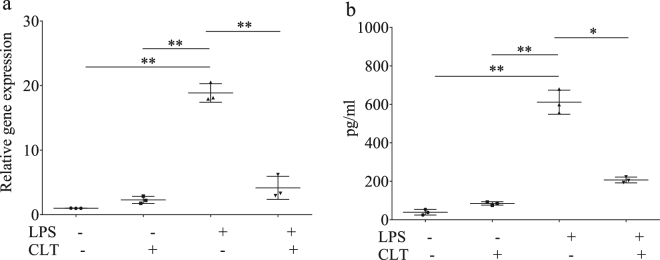
Clothianidin inhibits *TNF-α* expression induced by LPS treatment. In human THP-1 cells *TNF-α* transcription rate was measured by qRT-PCR (**a**) and TNF-α secreted protein in cell free supernatant by ELISA (**b**), after overnight incubation with Clothianidin (100 ng/ml), followed by LPS stimulation for 1 h (1 µg/ml), and compared with values obtained in untreated cells or exposed to Clothianindin but left unchallenged. Data are reported as mean ± SEM and are representative of 3 independent experiments, with 3 replicates each (One-Way ANOVA, all p < 0.05).

Insect immune response is negatively modulated by Clothianidin, which disrupts NF-κB signaling by up-regulating a negative modulator of this transcription factor^14^. Because NF-κB signaling underpins the modulation of several immune reactions in animals, we wanted to assess if this alteration induced by Clothianidin occurred also in humans. To test this hypothesis, we focused our attention on the effect of Clothianidin on the expression profile of the Tumor Necrosis Factor Alpha (TNF-α), a pro-inflammatory cytokine regulated by NF-κB via TLR-4^23^, both at transcriptional and translational level, using an immune cell line (THP-1), which expresses the nicotinic acetylcholine receptor^24^. Our data clearly indicate that exposure to Clothianidin disrupts the LPS-mediated induction of TNF-α expression, both in terms of transcript level (Fig. 1a) (One-Way ANOVA: F = 148.09; df = 3; p < 0.001) and protein production (Fig. 1b) (One-Way ANOVA: F = 183.61; df = 3; p < 0.001). The experimental concentration of Clothianidin used (100 ng/ml) did not have any cytotoxic effect on THP1 cells, as demonstrated by lactate dehydrogenase (LDH) release across a range of different doses of this insecticide (Fig. S1). Collectively, these results demonstrate that Clothianidin inhibits TNF-α expression, which is under NF-κB control.

**Figure 2:**
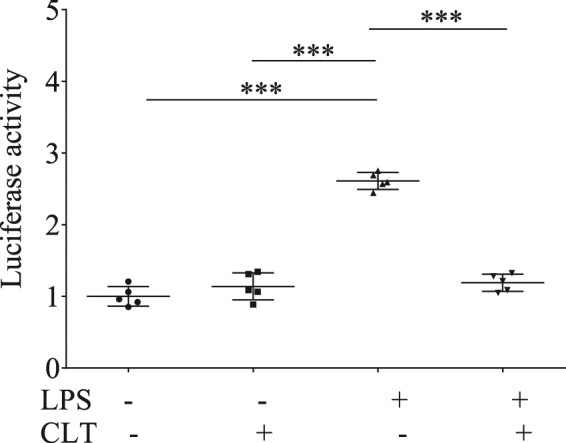
Clothianidin inhibits the expression of a NF-κB responsive reporter gene. NF-κB induction as affected by insecticide exposure was measured in human THP-1 cells, using a NF-κB luciferase reporter after incubation with Clothianidin overnight (100 ng/ml), followed by LPS stimulation for 1 h (1 µg/ml), and compared with values obtained in untreated cells or exposed to Clothianidin but left unchallenged. Data are reported as mean ± SEM and are representative of 3 independent experiments, with 5 replicates each (One-Way ANOVA, all p < 0.05).

To unequivocally demonstrate that Clothianidin exposure interferes with NF-κB activation, we stably transfected the THP-1 cell line with lentiviral particles carrying a NF-κB-responsive luciferase-expressing reporter gene (CignalLentiReporters, SABiosciences). The cells were incubated overnight, in presence or absence of Clothianidin, at the same concentration indicated above, and then treated with LPS or left unchallenged. When LPS challenge was performed in presence of Clothianidin, a significant (One-Way ANOVA: F = 137.09; df = 3; p < 0.001) inhibition of LPS-induced enhancement of the reporter gene expression was observed, indicating the occurrence of a negative effect of this neonicotinoid insecticide on NF-κB signaling (Fig. 2).

### Clothianidin alters the transcriptome of an immune cell line

**Figure 3:**
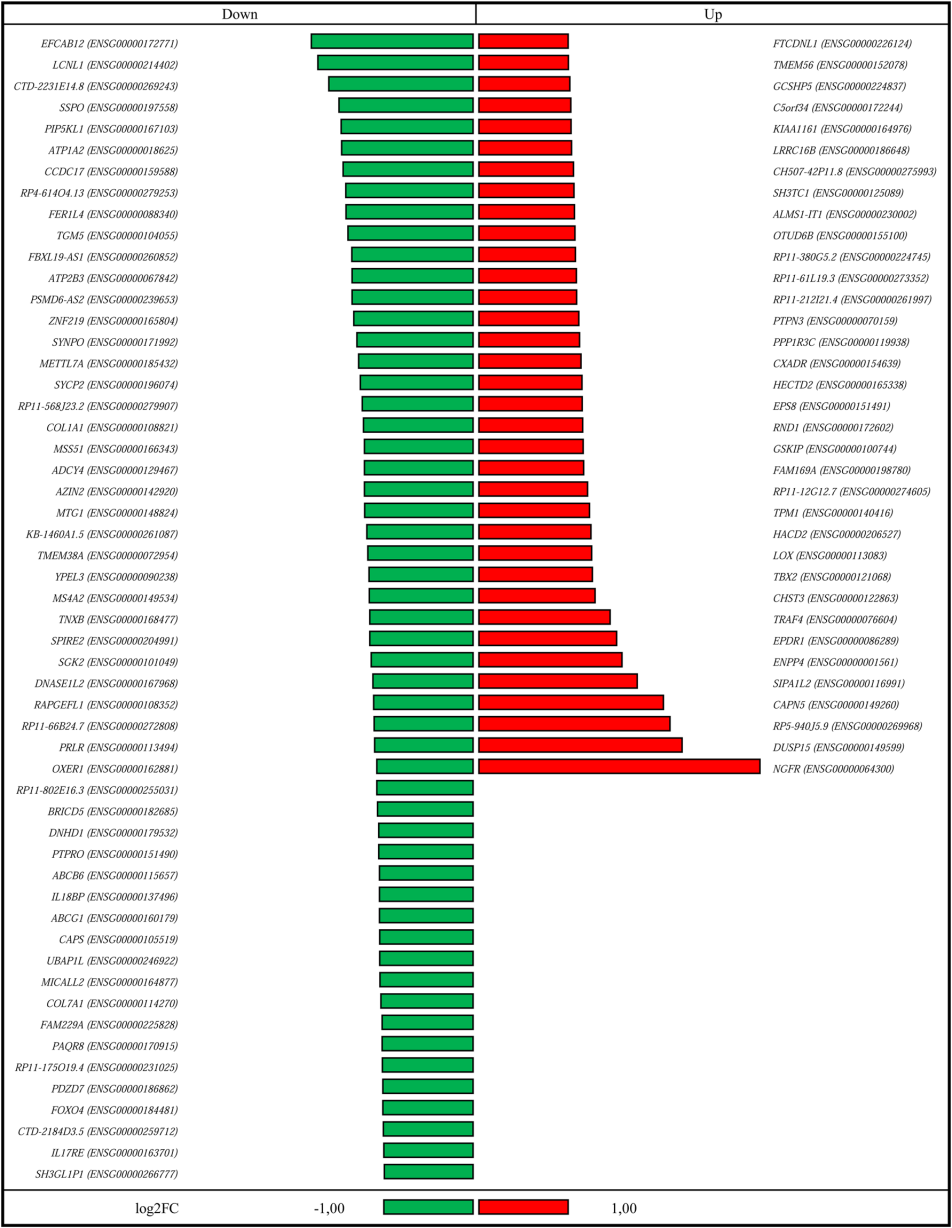
List of significantly (FDR <0.05) up- and down-regulated genes with Log_2_ fold change higher than 1 and lower than −1, respectively.

In order to identify the molecular networks underlying the inhibition of NF-κB activation induced by Clothianidin, we performed a transcriptomic analysis by RNA-Seq of the human THP-1 cell line exposed overnight to this neonicotinoid, at a concentration of 100 ng/ml, the same used in the experiments described above. After trimming and quality control of the obtained sequences, about 97% resulted as high quality reads (Supplementary Table S1), and were mapped against *Homo sapiens* reference genome (Ensembl, GRCh38). Principal component analysis (PCA) was applied to the dataset showing two distinct clusters, confirming replicate uniformity (Supplementary Figure S2). Differential expression analysis by false discovery rate (FDR) (P < 0.05) showed that 2,833 and 2,678 genes were significantly up- and down-regulated, respectively, in Clothianidin treated cells (Supplementary Figure S3 a, b). To select the most differentially expressed genes, we applied a more stringent filter for Log2 fold change of >+1 or <−1, which allowed the identification of 36 genes up-regulated and 54 down-regulated; both categories included immune genes under NF-κB transcriptional control, such as TNF receptor-associated factor 4 (TRAF4), TNF receptor-associated factor 6 (TRAF6), Fork head box protein O4 (FOXO4), Interleukin-18-binding protein(IL18BP) and Interleukin-17 receptor (IL17R) (Fig. 3). The concurrent up-regulation of the negative modulator of NF-κB activation TRAF4 and the down-regulation of TRAF6, exerting an opposite activity, well account for the reduced expression of genes controlled by this transcription factor. The entire collection of raw data is available on public database with BioProject Number PRJNA392257 (National Center of Biotechnology Information, U.S. National Library).

To analyze the putative interactions among proteins encoded by genes found to be differentially expressed and, then, of particular functional importance to face the stress induced by Clothianidin exposure, we used the String software^25^. This approach allowed the identification of a network of interactions among nerve growth factor receptor (NGFR), TRAF4 and TRAF6, which indicate how the core alteration of NF-κB signaling in the immune cell line THP-1 induced by Clothianidin is associated with a transcriptional change related to neural functions.

**Figure 4:**
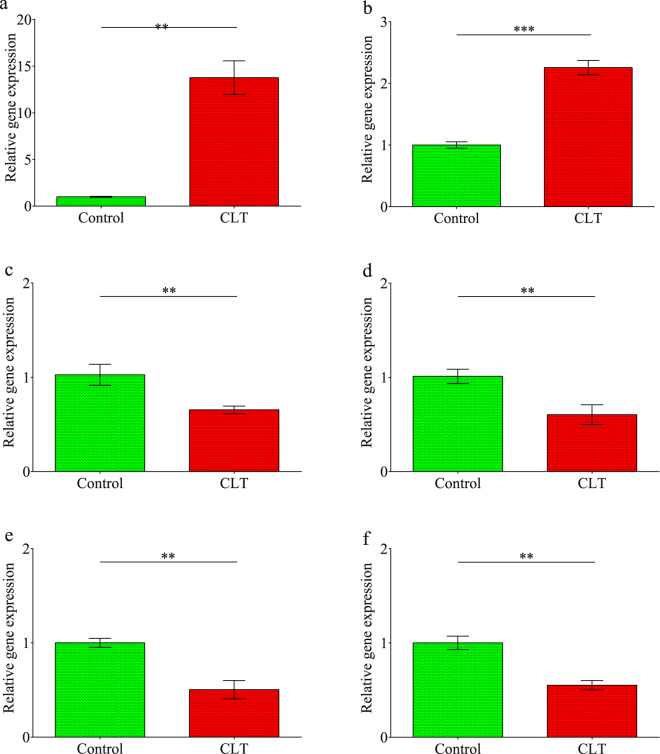
Relative gene expression in THP-1 cell treated with Clothianidin: NGFR (**a**); TRAF4 (**b**); TRAF6 (**c**); FOXO4 (**d**); IL18BP (**e**); IL17R (**f**). Data are reported as a mean ± SEM of 3 independent experiments, with 5 replicates each. (Student’s t test, all p < 0.05).

To validate the RNA-Seq analysis, the expression of the immune-related genes, found to be markedly modulated, was further assessed in an independent qRT-PCR experiment, where THP-1 cells were treated with Clothianidin overnight, at the same concentration used for RNA-Seq analysis. TRAF4 and NGFR were significantly up-regulated (Student’s t test: TRAF4, t = −17.064, df = 4, P < 0.001; NGFR, t = −12.275, df = 2, P = 0.007), while TRAF6, FOXO4, IL18BP and IL17R were down-regulated, as expected on the basis of RNA-Seq analysis (Student’s t test: TRAF6, t = 5.438, df = 4, P = 0.006; FOXO4,t = 5.444, df = 4, P = 0.006; IL18BPt = 7.995, df = 4, P = 0.001; IL17R,t = 8.976, df = 4, P = 0.001) (Fig. 4).

Collectively, these results allow to conclude that exposure to Clothianidin of the human cell line THP-1 determines a negative modulation of NF-κB signaling, associated with an up-regulation of TRAF4 and a down-regulation of TRAF6, a negative and a positive modulator, respectively, of NF-κB activation, which partly account for the observed immunosuppressive effects.

## Discussion

In this study we demonstrate that human THP-1 cells treated with the neonicotinoid insecticide Clothianidin react to an inflammatory stimulus by showing a lower expression of the cytokine TNF-α, due to a reduced activation of NF-κB, which controls its transcription. The negative impact of this neonicotinoid on NF-κB signaling has been recently reported in insects, and thought to be one of the stress elements that can contribute to the reduced efficacy of antiviral immune barriers controlling DWV replication in honeybees bearing covert infections of this viral pathogen^14^. The observed similar effects of Clothianidin on immune responses by cells of organisms in phylogenetically distant lineages indicate the occurrence of conserved mechanisms of cross-modulation between the nervous and immune system^26–28^. The nervous and the immune systems are traditionally thought to be separate functional entities and, as such, are separately studied. However, it is increasingly evident that this is not the case and their intimate interaction is a fascinating research area that continuously generates novel information on the subtle mechanisms involved^28^ and on their wide occurrence in the animal kingdom^27^. This conceptual framework nicely accounts for the observed immunomodulation by the acetylcholine agonist Clothianidin, even though the underlying molecular network that modulates this response remain still largely unexplored^14^. These conserved pathways of neuroimmune regulation and the fact that Clothianidin binds, even though with much lower affinity than in insects, to the human α4β2nicotinic acetylcholine receptor (α4β2AchR)^29^ were the major elements stimulating the present study, aiming to discover any immunotoxic effect that neonicotinoids may have on vertebrates.

Using STRING software, we highlighted a tight interaction between TRAF4, TRAF6 and the NGFR (Supplementary Figure S4). A previous study has reported that co-expression of NGFR with TRAF6 enhances expression of NF-κB, while TRAF4 negatively interferes with this process^30^. This further reinforces the tight relation between the nervous and immune systems. Indeed, the NGF in vertebrates can act as a homologue of the fly Toll ligand Spaetzle in eliciting immune reactions^31^, and its perception by THP-1 cells seems to be influenced by the acetylcholine agonist Clothianidin, which enhances the transcription of NGFR. Then, the observed transcriptional regulation may potentially influence cross-communication between the nervous and the immune systems^28^.

Our experimental data on THP-1 cells clearly indicate that exposure to Clothianidin is detrimental for the expression of genes under NF-κB control, as similarly observed in insects^14^. Indeed, both the RNA-Seq and qRT-PCR data revealed marked effects of Clothianidin exposure on the expression of genes linked to immune response. In particular, TRAF4 and TRAF6, which are members of the TRAF protein family, largely associated with the immune response^32^, resulted up-regulated and down-regulated, respectively. These proteins are involved in a number of transduction pathways of immune signaling molecules, with TRAF6 promoting NF-κB activation, while TRAF4 is a negative modulator of this transcription factor, as it competes for its binding sites on signal transduction proteins recruiting TRAF6^33^. The concurrent transcriptional down-regulation of IL17R induced by Clothianidin, associated with the TRAF6 and TRAF4 changes mentioned above, suggest that IL17 immune signaling is negatively influenced by neonicotinoids in THP-1 cells. Moreover, we can assume the occurrence of similar immune disruption pathways, largely driven by the same mechanism, that can be further aggravated by the down-regulation of proteins involved in NF-κB activation, such as IL18BP^34^. In contrast, the down-regulation of FOXO4 is not easy to interpret. This transcription factor is a member of the FOXO protein family, which is central in the integration of growth factor signaling, oxidative stress and inflammation^35^. Recent work has demonstrated that knockdown of FOXO4 does not affect NF-κB activation, suggesting that FOXO4 acts downstream in the signaling pathway^36^. Then, its down-regulation by Clothianidin treatment could further reinforce the inhibition of NF-κB activation. This is a likely hypothesis that merits to be investigated. Future studies will have to address these intriguing neuroimmune pathways, which are poorly known, in particular in non-vertebrate model systems, in order to fully appreciate the whole impact of neurotoxic molecules on the immune system. This is an important research area that is currently not adequately considered in toxicological studies.

In conclusion, our data show that the insecticide Clothianidin negatively influences in a human cell line the expression of immune related genes, under control of the transcription factorNF-κB, as similarly observed in insects. These findings are interesting, but their relevance will have to be assessed at the organism level to see if they may represent a significant threat for human health.

## Methods

### Cell culture

The human monocytic cell line THP-1 (ATCC number: TIB-202; LGC Standards GmbH) was cultured in RPMI 1640 (Life technologies) medium supplemented with 10% FCS (Life technologies) and 1 mM of L-glutamine (Life technologies), under 5% CO_2_ at 37 °C.

### Total RNA extraction and cDNA Synthesis

Total RNA extraction from THP- 1 cells was carried out by using TRIzol (ThermoScientific), according to the manufacturer’s instructions. The RNA yield and A260/280 ratio were monitored with a NanoDrop ND 100 spectrometer (NanoDrop Technologies), and RNA integrity was verified using the 2100 Bioanalyzer (Agilent Technologies). cDNA synthesis was carried out starting from 1 μg of total RNA and using the High Capacity cDNA Reverse Transcription Kit(ThermoScientific), according to the manufacturer’s protocol.

### RNA-Seq

An amount of 10^6^/well cells (control and Clothianidin treated, 3 biological replicates each) were processed for RNA-Seq analysis. Indexed libraries were prepared using 1 µg of each RNA purified with TruSeq Stranded mRNA Sample Prep Kit (Illumina), according to the manufacturer’s instructions. Libraries were quantified using the Agilent 2100 Bioanalyzer (Agilent Technologies) and pooled, so that each index-tagged sample was present in equimolar amounts, with a final concentration of the pooled samples of 2 nM. The pooled samples were subjected to cluster generation and sequencing, using an Illumina HiSeq. 2500 System (Illumina) in a 2 × 100 paired-end format, at a final concentration of 8 pmol. The raw sequence files generated (.fastq files) underwent quality control analysis, using FastQC (http://www.bioinformatics.babraham.ac.uk/projects/fastqc/).

Raw reads were checked for quality with FastQC v0.11.3 and then trimming and removal of adapters were performed with Trimmomatic v0.33 (minimum quality score 25, minimum length 35). The obtained reads were then mapped against *Homo sapiens* reference genome (Ensembl, GRCh38) with STAR (v2.5.0b), providing the reference gene annotation file with known transcripts. FeatureCounts (v1.4.5-p1) was used to perform read summarization at gene level, with the strand-specific option “reversely stranded”. Statistical analysis of the read counts was performed with R, using the HTSFilter package, to remove low expressed genes, and the NOIseq package, to perform differential expression analysis. Gene Ontology enrichment analysis of the differentially expressed genes was performed with the GOStat package.

### qRT-PCR

The expression profile of the immune genes that showed the most pronounced transcriptional regulation was also analyzed by TaqManqRT-PCR, using specific primers and probes:*TNF-α* (AssayID: Hs00174128_m1), *FOXO4* (Assay ID: Hs00172973_m1), *NGFR* (Assay ID: Hs00609976_m1), *IL18BP* (Assay ID: Hs00271720_m1), *TRAF4* (Assay ID: Hs01030628_g1), *TRAF6* (Assay ID: Hs00939742_g1) *and IL17R* (Assay ID: Hs01056316_m1). All probes were normalized to *Gapdh* (Assay ID: Hs02786624_g1) as internal control (Applied Biosystems). All fold changes were calculated using the ΔΔCt method (Livack *et al*., 2001) and compared with untreated cell. Amplifications were performed with ABI PRISM 7900HT (Applied Biosystems).

### Clothianidin effect on TNF-α expression

Clothianidin was obtained from Sigma (Cat No: 33589) and used as follows: 10^6^/well cells were seeded in a 24 well plate and were pre-treated with Clothianidin (100 ng/ml) overnight, then, stimulated for 1 hour with LPS 1 μg/ml (Sigma), and compared with unchallenged cells and untreated basal controls. Experimental cells were washed in PBS before RNA extraction. A qRT-PCR analysis was performed to measure the transcription rate of TNF-α gene in THP-1 cell treated as described above. TNF-α protein secretion was measured in cell free supernatant using TNF-α DuoSet ELISA development kit (R&D system), following manufacturer’s procedure.

### NF-κB reporter gene assay

THP-1 cells were infected with 10 µL lentiviral particles carrying a NF-κB responsive luciferase-expressing reporter gene (CignalLenti Reporters, SABiosciences), according to the protocol provided by the manufacturer, followed by selection with puromycin. Once the cell line was established, THP-1 were incubated with Clothianidin (100 ng/ml) overnight and then treated with LPS (1 μg/ml) for 1 hour. NF-κB activity was measured using Dual Glo Luciferase assay (Promega), according to the manufacturer’s procedure. Luciferase activity was normalized for all samples with total amount of proteins.

### Cytotoxicity assay

THP-1 cell were treated overnight with different dose of Clothianidin and cytotoxicity was evaluated by measuring lactate dehydrogenase (LDH) release in the supernatant, using a CytoTox 96^®^ Non-Radio cytotoxicity assay kit (Promega, Madison, WI, USA), according to the manufacturer’s instructions.

### Statistical analysis

Normality of data was checked with Shapiro-Wilk test, while homoscedasticity was tested with Levene’s procedure. Differences in the relative expression of TNF-α, secreted TNF-α protein and Luciferase activity of the NF-κB responsive reporter gene were analyzed with One-Way ANOVA followed by Games-Howell post-hoc test (parametric and non-homoscedastic procedure).

Two-tailed parametric non-homoscedastict-test was used to analyze differences in relative expression of NGFR, while for TRAF4, TRAF6, FOXO4, IL18BP and IL17R gene expression was analyzed with the two-tailed parametric homoscedastic t-test. Differences in the LDH amounts released by cells exposed to Clothianidin were analysed by One-Way ANOVA, followed by LSD post-hoc test (parametric and homoscedastic procedure). These analyses were performed by using Prism v.5 for Mac OSX (GraphPad software, San Diego, CA, USA). All statistical data are available in the Supplementary Table 2.

## Electronic supplementary material

Supplementary information

## References

[1] Simon-Delso N (2015). Systemic insecticides (neonicotinoids and fipronil): trends, uses, mode of action and metabolites. Environ Sci Pollut Res Int.

[2] Tomizawa M, Casida JE (2005). Neonicotinoid insecticide toxicology: mechanisms of selective action. Annu Rev Pharmacol Toxicol.

[3] Elbert A, Haas M, Springer B, Thielert W, Nauen R (2008). Applied aspects of neonicotinoid uses in crop protection. Pest Manag Sci.

[4] EASAC. Ecosystem services, agriculture and neonicotinoids. *EASAC policy report* **26** (2015).

[5] Pistorius, J., Bischoff, G., Heimbach, U. & Stähler, M. Bee poisoning incidents in Germany in spring 2008 caused by abrasion of active substance from treated seeds during sowing of maize. *ulius-Kühn-Archiv* **423** (2009).

[6] Yang EC, Chuang YC, Chen YL, Chang LH (2008). Abnormal foraging behavior induced by sublethal dosage of imidacloprid in the honey bee (Hymenoptera: Apidae). J Econ Entomol.

[7] Han P, Niu CY, Lei CL, Cui JJ, Desneux N (2010). Use of an innovative T-tube maze assay and the proboscis extension response assay to assess sublethal effects of GM products and pesticides on learning capacity of the honey bee Apis mellifera L. Ecotoxicology.

[8] Henry M (2012). A common pesticide decreases foraging success and survival in honey bees. Science.

[9] Alaux C (2010). Interactions between Nosema microspores and a neonicotinoid weaken honeybees (Apis mellifera). Environmental Microbiology.

[10] Aufauvre J (2012). Parasite-insecticide interactions: a case study of Nosema ceranae and fipronil synergy on honeybee. Scientific Reports.

[11] Pettis JS, vanEngelsdorp D, Johnson J, Dively G (2012). Pesticide exposure in honey bees results in increased levels of the gut pathogen Nosema. Naturwissenschaften.

[12] Fauser-Misslin A, Sadd BM, Neumann P, Sandrock C (2014). Influence of combined pesticide and parasite exposure on bumblebee colony traits in the laboratory. Journal of Applied Ecology.

[13] Doublet V, Labarussias M, de Miranda JR, Moritz RF, Paxton RJ (2015). Bees under stress: sublethal doses of a neonicotinoid pesticide and pathogens interact to elevate honey bee mortality across the life cycle. Environ Microbiol.

[14] Di Prisco G (2013). Neonicotinoid clothianidin adversely affects insect immunity and promotes replication of a viral pathogen in honey bees. Proc Natl Acad Sci USA.

[15] Brandt A (2016). The neonicotinoids thiacloprid, imidacloprid, and clothianidin affect the immunocompetence of honey bees (Apis mellifera L.). J Insect Physiol.

[16] Nazzi F (2012). Synergistic parasite-pathogen interactions mediated by host immunity can drive the collapse of honeybee colonies. PLoS Pathog.

[17] Nazzi F, Pennacchio F (2014). Disentangling multiple interactions in the hive ecosystem. Trends Parasitol.

[18] Di Prisco G (2016). A mutualistic symbiosis between a parasitic mite and a pathogenic virus undermines honey bee immunity and health. Proc Natl Acad Sci USA.

[19] Sánchez-Bayo F (2016). Are bee diseases linked to pesticides? — A brief review. Environment International.

[20] Spatuzza C (2008). Physical and functional characterization of the genetic locus of IBtk, an inhibitor of Bruton’s tyrosine kinase: evidence for three protein isoforms of IBtk. Nucleic Acids Res.

[21] Cimino AM, Boyles AL, Thayer KA, Perry MJ (2017). Effects of Neonicotinoid Pesticide Exposure on Human Health: A Systematic Review. Environmental Health Perspectives.

[22] Seltenrich N (2017). Catching Up with Popular Pesticides: More Human Health Studies Are Needed on Neonicotinoids. Environ Health Perspect.

[23] Akira S, Takeda K (2004). Toll-like receptor signalling. Nat Rev Immunol.

[24] Costantini TW (2015). A Human-Specific alpha7-Nicotinic Acetylcholine Receptor Gene in Human Leukocytes: Identification, Regulation and the Consequences of CHRFAM7A Expression. Mol Med.

[25] Szklarczyk D (2015). STRINGv10: protein-protein interaction networks, integrated over the tree of life. Nucleic Acids Res.

[26] Tracey KJ (2009). Reflex control of immunity. Nat Rev Immunol.

[27] Tracey KJ (2011). Ancient Neurons Regulate Immunity. Science (New York, N.Y.).

[28] Talbot S, Foster SL, Woolf CJ (2016). Neuroimmunity: Physiology and Pathology. Annu Rev Immunol.

[29] Li P, Ann J, Akk G (2011). Activation and modulation of human alpha4beta2 nicotinic acetylcholine receptors by the neonicotinoids clothianidin and imidacloprid. J Neurosci Res.

[30] Ye X (1999). TRAF family proteins interact with the common neurotrophin receptor and modulate apoptosis induction. J Biol Chem.

[31] Hepburn L (2014). A Spaetzle-like role for Nerve Growth Factor β in vertebrate immunity to Staphylococcus aureus. Science (New York, N.Y.).

[32] Aggarwal K, Silverman N (2008). Positive and negative regulation of the Drosophila immune response. BMB Rep.

[33] Xie P (2013). TRAF molecules in cell signaling and in human diseases. J Mol Signal.

[34] Dinarello CA, Fantuzzi G (2003). Interleukin-18 and host defense against infection. J Infect Dis.

[35] Hedrick, S. M., Michelini, R. H., Doedens, A. L., Goldrath, A. W. & Stone, E. L. FOXO transcription factors throughout T cell biology. *Nature reviews. Immunology* **12**, 10.1038/nri3278 (2012). 10.1038/nri3278 PMC3875397 22918467

[36] Oteiza A, Mechti N (2015). Control of FoxO4 Activity and Cell Survival by TRIM22 Directs TLR3-Stimulated Cells Toward IFN Type I Gene Induction or Apoptosis. J Interferon Cytokine Res.

