# Supplementary material for: The neonicotinoid insecticide Clothianidin adversely affects immune signaling in a human cell line
Source: Sci Rep. 2017 Oct 18;7:13446. doi: 10.1038/s41598-017-13171-z (PMC5647381; doi:10.1038/s41598-017-13171-z)
Supplement: Supplementary file 1 — Supplementary information [file 41598_2017_13171_MOESM1_ESM.doc]

**Supplementary Information**

**The neonicotinoid insecticide Clothianidin adversely affects immune signaling in a human cell line**

Gennaro Di Prisco, Marco Iannaccone, Flora Ianniello, Rosalba Ferrara, Emilio Caprio, Francesco Pennacchio, Rosanna Capparelli

Department of Agricultural Sciences, University of Napoli “Federico II” – Via Università 100, 80055 Portici, Napoli, Italy.

**Table S1.** Quality control of reads after trimming.

| **Sample** | **Species** | **Reads before data quality control** | **Reads after data quality control** | **%** |
| --- | --- | --- | --- | --- |
| CLT_1 | *H.sapiens* | 11380542 | 11024876 | 96,87479 |
| CLT_2 | *H.sapiens* | 16463406 | 16012808 | 97,26303 |
| CLT_3 | *H.sapiens* | 16787304 | 16343923 | 97,35883 |
| THP1_1 | *H.sapiens* | 25140542 | 24534971 | 97,59126 |
| THP1_2 | *H.sapiens* | 15651170 | 15349298 | 98,07125 |
| THP1_3 | *H.sapiens* | 16758945 | 16420919 | 97,98301 |
|  |  |  |  |  |
|  | | | |  |

**Table S2.** Output of statistical analyses.

| **Figure 1A** | | |  |  |  |  |
| --- | --- | --- | --- | --- | --- | --- |
| **Shapiro-Wilk test** | | |  |  |  |  |
| **Treatment** | **Statistic** | **df** | **Sig.** |  |  |  |
| LPS- CLT- | .946 | 3 | .551 |  |  |  |
| LPS- CLT+ | .987 | 3 | .784 |  |  |  |
| LPS+ CLT- | .805 | 3 | .126 |  |  |  |
| LPS+ CLT+ | .830 | 3 | .187 |  |  |  |
|  |  |  |  |  |  |  |
| **Levene's test** | | | |  |  |  |
| **F** | **df1** | **df2** | **Sig.** |  |  |  |
| 6.735 | 3 | 8 | .014 |  |  |  |
|  |  |  |  |  |  |  |
| **One-Way ANOVA** | | | | | |  |
|  | **Sum of square** | **df** | **MeanSquare** | **F** | **Sig.** |  |
| BetweenGroups | 618.052 | 3 | 206.017 | 148.093 | <.001 |  |
| WithinGroups | 11.129 | 8 | 1.391 |  |  |  |
| Total | 629.181 | 11 |  |  |  |  |
|  |  |  |  |  |  |  |
| **Games-Howell post hoc test** | | | | | | |
| **Comparisons** | | **Meandifference (I-J)** | **Std. Error** | **Sig.** | **95% Confidenceinterval** | |
| Treatment (i) | Treatment (j) |  |  |  | Lower Bound | UpperBound |
| LPS- CLT- | LPS- CLT+ | -1.287 | .3227 | .139 | -3.519 | .944 |
| LPS- CLT- | LPS+ CLT- | -17.852 | .8292 | .005 | -23.595 | -12.109 |
| LPS- CLT- | LPS+ CLT+ | -3.161 | 1.031 | .217 | -1.304 | 3.982 |
| LPS- CLT+ | LPS- CLT- | 1.287 | .3227 | .139 | -.944 | 3.519 |
| LPS- CLT+ | LPS+ CLT- | -16.565 | .890 | .002 | -21.362 | -11.768 |
| LPS- CLT+ | LPS+ CLT+ | -1.874 | 1.080 | .463 | -8.129 | 4.381 |
| LPS+ CLT- | LPS- CLT- | 17.852 | .829 | .005 | 12.109 | 23.595 |
| LPS+ CLT- | LPS- CLT- | 16.565 | .890 | .002 | 11.768 | 21.362 |
| LPS+ CLT- | LPS+ CLT+ | 14.691 | 1.323 | .002 | 9.179 | 2.203 |
| LPS+ CLT+ | LPS- CLT- | 3.161 | 1.031 | .217 | -3.982 | 1.304 |
| LPS+ CLT+ | LPS- CLT+ | 1.874 | 1.080 | .463 | -4.381 | 8.129 |
| LPS+ CLT+ | LPS+ CLT- | -14.691 | 1.323 | .002 | -2.203 | -9.179 |

| **Figure 1B** | | |  |  |  |  |
| --- | --- | --- | --- | --- | --- | --- |
| **Shapiro-Wilk test** | | |  |  |  |  |
| **Treatment** | **Statistic** | **df** | **Sig.** |  |  |  |
| LPS- CLT- | .996 | 3 | .886 |  |  |  |
| LPS- CLT+ | .947 | 3 | .554 |  |  |  |
| LPS+ CLT- | .968 | 3 | .654 |  |  |  |
| LPS+ CLT+ | .977 | 3 | .708 |  |  |  |
|  |  |  |  |  |  |  |
| **Levene's test** | | | |  |  |  |
| **F** | **df1** | **df2** | **Sig.** |  |  |  |
| 4.173 | 3 | 8 | .047 |  |  |  |
|  |  |  |  |  |  |  |
| **One-Way ANOVA** | | | | | |  |
|  | **Sum of square** | **df** | **MeanSquare** | **F** | **Sig.** |  |
| BetweenGroups | 609583.330 | 3 | 203194.440 | 183.61 | <.001 |  |
| WithinGroups | 8853.330 | 8 | 1106.670 |  |  |  |
| Total | 618436.670 | 11 |  |  |  |  |
|  |  |  |  |  |  |  |
| **Games-Howell post hoc test** | | | | | | |
| **Comparisons** | | **Meandifference (I-J)** | **Std. Error** | **Sig.** | **95% Confidenceinterval** | |
| Treatment (i) | Treatment (j) |  |  |  | Lower Bound | UpperBound |
| LPS- CLT- | LPS- CLT+ | -45,667 | 9,787 | ,047 | -90,194 | -1,140 |
| LPS- CLT- | LPS+ CLT- | -572,000 | 37,058 | ,007 | -802,428 | -341,571 |
| LPS- CLT- | LPS+ CLT+ | -167,667 | 12,129 | ,001 | -217,089 | -118,244 |
| LPS- CLT+ | LPS- CLT- | 45,667 | 9,787 | ,047 | 1,140 | 90,194 |
| LPS- CLT+ | LPS+ CLT- | -526,333 | 36,448 | ,010 | -768,450 | -284,216 |
| LPS- CLT+ | LPS+ CLT+ | -122,000 | 10,110 | ,003 | -168,778 | -75,222 |
| LPS+ CLT- | LPS- CLT- | 572,000 | 37,058 | ,007 | 341,571 | 802,428 |
| LPS+ CLT- | LPS- CLT- | 526,333 | 36,448 | ,010 | 284,216 | 768,450 |
| LPS+ CLT- | LPS+ CLT+ | 404,333 | 37,145 | ,015 | 175,369 | 633,297 |
| LPS+ CLT+ | LPS- CLT- | 167,667 | 12,129 | ,001 | 118,244 | 217,089 |
| LPS+ CLT+ | LPS- CLT+ | 122,000 | 10,110 | ,003 | 75,222 | 168,778 |
| LPS+ CLT+ | LPS+ CLT- | -404,333 | 37,145 | ,015 | -633,297 | -175,369 |

| **Figure 2** | | |  |  |  |  |
| --- | --- | --- | --- | --- | --- | --- |
| **Shapiro-Wilk test** | | |  |  |  |  |
| **Treatment** | **Statistic** | **df** | **Sig.** |  |  |  |
| LPS- CLT- | 1.00 | 3 | .985 |  |  |  |
| LPS- CLT+ | .915 | 3 | .433 |  |  |  |
| LPS+ CLT- | .818 | 3 | .159 |  |  |  |
| LPS+ CLT+ | .790 | 3 | .091 |  |  |  |
|  |  |  |  |  |  |  |
| **Levene's test** | | | |  |  |  |
| **F** | **df1** | **df2** | **Sig.** |  |  |  |
| .792 | 3 | 16 | .516 |  |  |  |
|  |  |  |  |  |  |  |
| **Onw-Way ANOVA** | | | | | |  |
|  | **Sum of square** | **df** | **MeanSquare** | **F** | **Sig.** |  |
| BetweenGroups | 8.545 | 3 | 2.848 | 137.090 | <.001 |  |
| WithinGroups | 52.957 | 16 | .021 |  |  |  |
| Total | 8.877 | 19 |  |  |  |  |
|  |  |  |  |  |  |  |
| **Games-Howell post hoc test** | | | | | | |
| **Comparisons** | | **Meandifference (I-J)** | **Std. Error** | **Sig.** | **95% Confidenceinterval** | |
| Treatment (i) | Treatment (j) |  |  |  | Lower Bound | UpperBound |
| LPS- CLT- | LPS- CLT+ | -.138 | .1045 | .577 | -.480 | .204 |
| LPS- CLT- | LPS+ CLT- | -1.610 | .081 | .000 | -1.872 | -1.348 |
| LPS- CLT- | LPS+ CLT+ | -.190 | .082 | .173 | -.452 | .073 |
| LPS- CLT+ | LPS- CLT- | .138 | .104 | .577 | -.204 | .480 |
| LPS- CLT+ | LPS+ CLT- | -1.472 | .100 | .000 | -1.806 | -1.138 |
| LPS- CLT+ | LPS+ CLT+ | -.051 | .100 | .953 | -.385 | .283 |
| LPS+ CLT- | LPS- CLT- | 1.610 | .081 | .000 | 1.348 | 1.872 |
| LPS+ CLT- | LPS- CLT- | 1.472 | .100 | .000 | 1.138 | 1.806 |
| LPS+ CLT- | LPS+ CLT+ | 1.421 | .075 | .000 | 1.179 | 1.662 |
| LPS+ CLT+ | LPS- CLT- | .190 | .082 | .173 | -.0731 | .452 |
| LPS+ CLT+ | LPS- CLT+ | .051 | .100 | .953 | -.283 | .385 |
| LPS+ CLT+ | LPS+ CLT- | -1.421 | .0755 | .000 | -1.662 | -1.179 |

| **Figure 4** | | | | | | | | | | |
| --- | --- | --- | --- | --- | --- | --- | --- | --- | --- | --- |
|  | | **Levene's Test for Equality of Variances** | | **t-test for Equality of Means** | | | | | | |
| **Gene** | **Equal variance sassumed** | **F** | **Sig.** | **t** | **df** | **Sig.**  **(2-tailed)** | **Mean Difference** | **Std. Error Difference** | **95% Confidence Interval of the Difference** | |
|  |  |  |  |  |  |  |  |  | **Lower** | **Upper** |
| **NGFR** | Yes | 12,602 | .024 | -12,275 | 4,000 | <.001 | -12,763 | 1,040 | -15,649 | -9,876 |
|  | No |  |  | -12,275 | 2,004 | .007 | -12,763 | 1,040 | -17,228 | -8,297 |
| **TRAF4** | Yes | 1,792 | .252 | -17,064 | 4,000 | <.001 | -1,256 | .074 | -1,461 | -1,052 |
|  | No |  |  | -17,064 | 2,779 | .001 | -1,256 | .074 | -1,501 | -1,011 |
| **TRAF6** | Yes | 1,626 | .271 | 5,438 | 4,000 | .006 | .372 | .068 | .182 | .562 |
|  | No |  |  | 5,438 | 2,513 | .019 | .372 | .068 | .128 | .615 |
| **FOXO4** | Yes | .354 | .584 | 5,444 | 4,000 | .006 | .407 | .075 | .200 | .615 |
|  | No |  |  | 5,444 | 3,640 | .007 | .407 | .075 | .191 | .623 |
| **IL18BP** | Yes | 1,781 | .253 | 7,995 | 4,000 | .001 | .498 | .062 | .325 | .670 |
|  | No |  |  | 7,995 | 2,903 | .005 | .498 | .062 | .296 | .699 |
| **IL17R** | Yes | .746 | .436 | 8,976 | 4,000 | .001 | .449 | .050 | .310 | .588 |
|  | No |  |  | 8,976 | 3,576 | .001 | .449 | .050 | .303 | .595 |

| **Supplementary Figure 1** | | |  |  |  |  |
| --- | --- | --- | --- | --- | --- | --- |
| **Shapiro-Wilk test** | | |  |  |  |  |
| **Treatment** | **Statistic** | **df** | **Sig.** |  |  |  |
| THP-1 | .968 | 5 | .860 |  |  |  |
| LDH ctlr + | .914 | 5 | .493 |  |  |  |
| 10 ng | .987 | 5 | .967 |  |  |  |
| 50 ng | .903 | 5 | .429 |  |  |  |
| 100 ng | .766 | 5 | .042 |  |  |  |
| 1000 ng | .913 | 5 | .483 |  |  |  |
|  |  |  |  |  |  |  |
| **Levene's test** | | | |  |  |  |
| **F** | **df1** | **df2** | **Sig.** |  |  |  |
| 2.241 | 4 | 24 | .083 |  |  |  |
|  |  |  |  |  |  |  |
| **One-Way ANOVA** | | | | | |  |
|  | **Sum of square** | **df** | **MeanSquare** | **F** | **Sig.** |  |
| BetweenGroups | 1.455 | 5 | .2911 | 266.886 | <.001 |  |
| WithinGroups | .026 | 24 | .001 |  |  |  |
| Total | 1.482 | 29 |  |  |  |  |
|  |  |  |  |  |  |  |
| **LSD post hoc test** | | | | | | |
| **Comparisons** | | **Meandifference (I-J)** | **Std. Error** | **Sig.** | **95% Confidenceinterval** | |
| Treatment (i) | Treatment (j) |  |  |  | Lower Bound | UpperBound |
| THP-1 | LDH ctlr + | -.613 | .021 | .000 | -.656 | -.569 |
| THP-1 | 10 ng | -.010 | .021 | .650 | -.053 | .033 |
| THP-1 | 50 ng | -.034 | .021 | .115 | -.077 | .009 |
| THP-1 | 100 ng | -.020 | .021 | .343 | -.063 | .023 |
| THP-1 | 1000 ng | -.053 | .021 | .018 | -.096 | -.009 |
| LDH ctlr + | 10 ng | .603 | .021 | .000 | .560 | .646 |
| LDH ctlr + | 50 ng | .578 | .021 | .000 | .535 | .621 |
| LDH ctlr + | 100 ng | .592 | .021 | .000 | .549 | .635 |
| LDH ctlr + | 1000 ng | .560 | .021 | .000 | .517 | .603 |
| 10 ng | 50 ng | -.0246 | .021 | .250 | -.068 | .018 |
| 10 ng | 100 ng | -.0106 | .021 | .616 | -.054 | .032 |
| 10 ng | 1000 ng | -.0432 | .021 | .050 | -.086 | -.000 |
| 50 ng | 100 ng | .014 | .021 | .509 | -.029 | .057 |
| 50 ng | 1000 ng | -.0186 | .021 | .382 | -.062 | .024 |
| 100 ng | 1000 ng | -.0326 | .021 | .132 | -.076 | .010 |

**
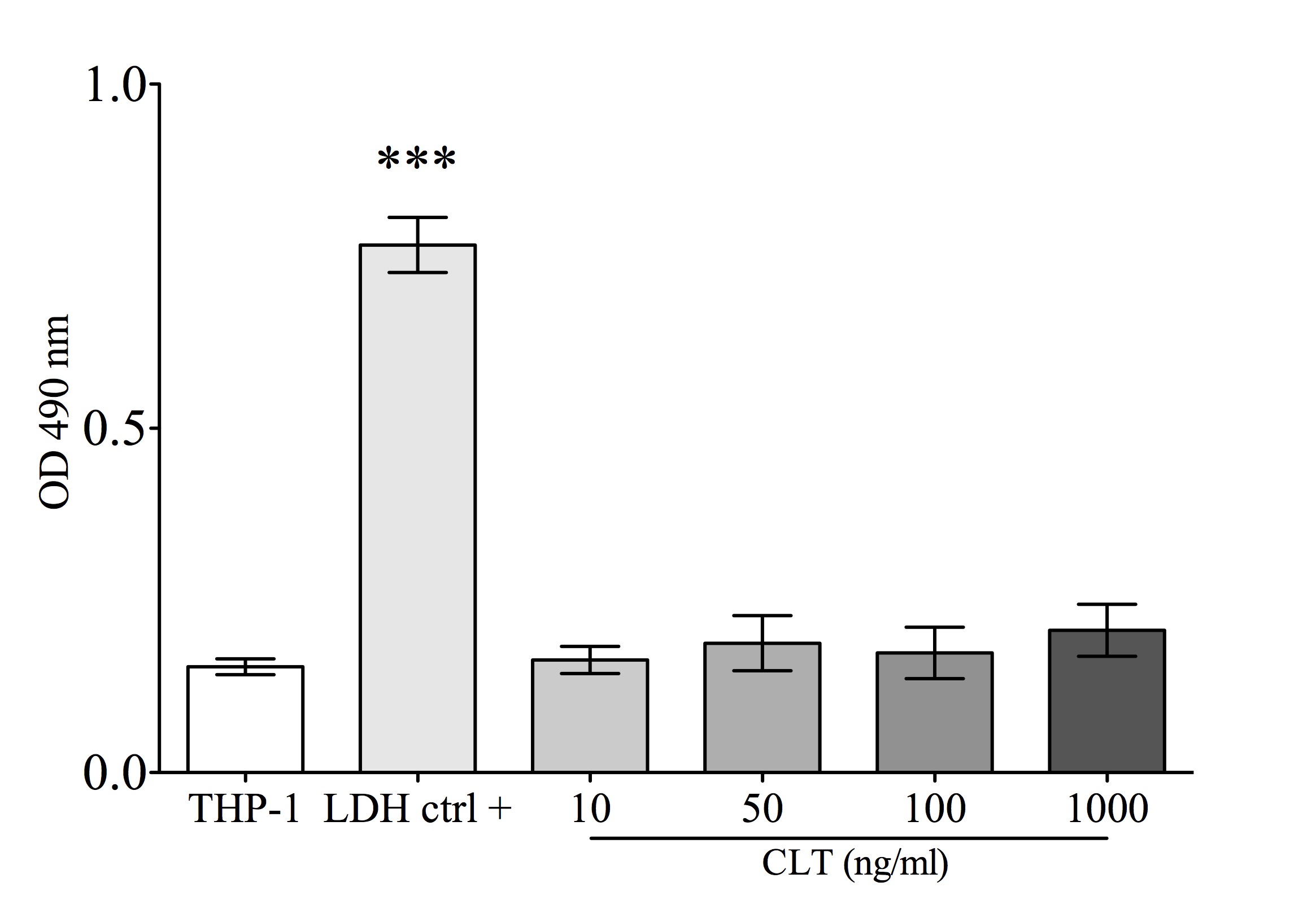
**

**Figure S1.** Cytotoxicity assay of Clothianidin. The occurrence of any cytotoxic effect exerted by Clothianidin (CLT) on human THP-1 cells was assessed by measuring the cellular release of lactate dehydrogenase (LDH), following overnight exposure to different CLT concentrations. All experimental doses induced responses significantly lower than in positive controls (LDH ctrl+), but not different among them. Data are reported as mean ± SEM and are representative of 3 independent experiments, with 5 replicates each (One-Way ANOVA, ***p<0.001).

**Figure S2.** PCA analysis shows the good overall quality of the experiment, as indicated by the high similarity between replicates.

a
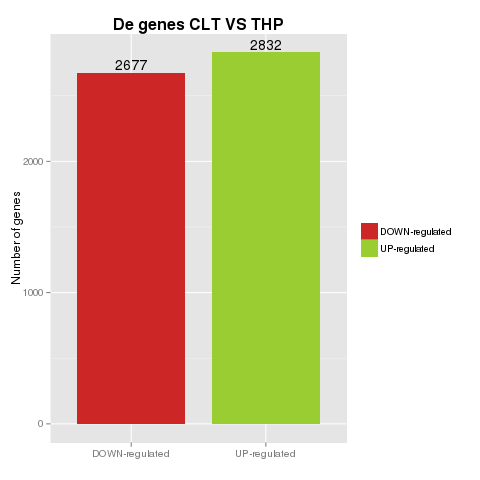
b
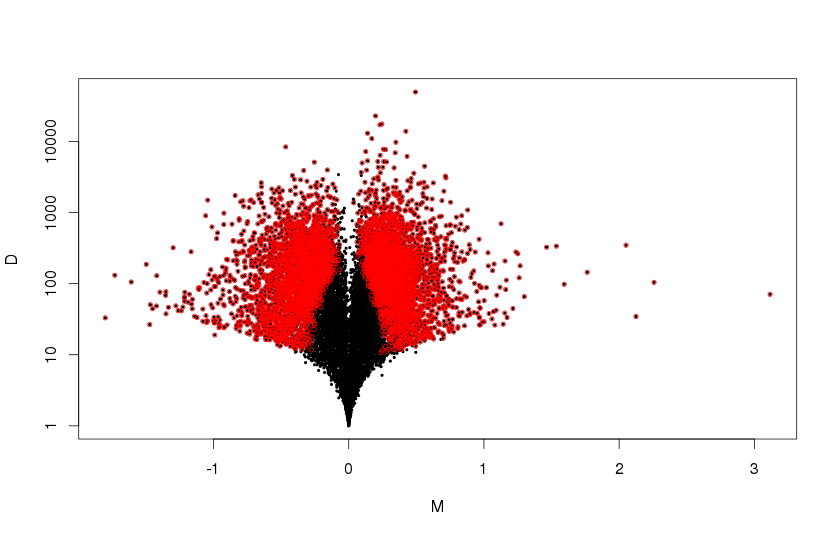


**Figure S3.** Number (a) and MD-plot (b) showing differentially expressed genes in THP-1 cell line treated with Clothianidin (M, Log-fold change and D, absolute value of the differences in expression between the two experimental conditions).


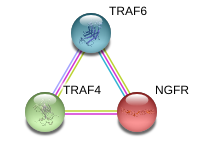


**Figure S4.** Interaction network of differentially expressed genes in THP-1 cell line treated with Clothianidin. Different line colors indicate the type of available evidence for the putative associations: green line, neighborhood evidence; purple line, experimental evidence; blue line, co-occurrence evidence; light-blue line, database evidence.
